# Effect of Mg on Plasticity and Microstructure of Al-Mg-Ga-Sn-In Soluble Aluminum Alloy

**DOI:** 10.3390/ma17215287

**Published:** 2024-10-30

**Authors:** Ning Ma, Jianfeng Zhu, Ke Chang, Yuxing Qin

**Affiliations:** 1 Shaanxi Key Laboratory of GreenPreparation and Functionalization for Inorganic Materials, School of Materials Science and Engineering, Shaanxi University of Science & Technology, Xi’an 710021, China; sweetmaning@126.com (N.M.); 210212129@sust.edu.cn (Y.Q.); 2 Xi’an Hantang Analysis and Test Co., Ltd., Northwest Institute for Nonferrous Metal Research, Xi’an 710016, China; ke24264@163.com

**Keywords:** Al-Mg-Ga-Sn-In soluble aluminum, Mg element, mechanical performance, dissolution

## Abstract

Soluble aluminum alloy materials used in underground operational tools are synthesized via a high-temperature smelting process. The microstructure and composition distribution of the alloy were analyzed using X-ray diffraction, metallographic microscopy, and scanning electron microscopy with energy-dispersive X-ray and electron backscatter diffraction techniques. The mechanical properties were evaluated using a universal testing machine and hardness tester, while solubility assessments were conducted in a constant-temperature water bath. This study focuses on the plasticity and dissolution characteristics of Al-Mg-Ga-Sn-In alloys with varying Mg contents. The tensile strength (σ_b_) of the alloy was 181.99 MPa, with an elongation (δ) of 27.49% and cross-sectional shrinkage (φ) of 11.67% at a magnesium content of 3.0 wt.%. Additionally, in the compressive test, the compressive yield strength (σ_sc_) was recorded at 188.32 MPa, while the compression rate (δ) was 27.06% and the section expansion rate (φ) was 138.66%. Furthermore, the alloy demonstrated the ability to dissolve spontaneously in water at 90 °C, exhibiting an average dissolution rate of 1.0 g·h^−1^cm^−2^ and a maximum dissolution rate of 3.25 g·h^−1^cm^−2^ after 12.0 h. Consequently, this alloy composition not only satisfies the requirements for rapid solubility but also exhibits favorable plasticity, providing a novel reference for the selection of soluble aluminum alloy materials.

## 1. Introduction

Soluble aluminum alloys play an irreplaceable and important role as downhole containment tools, which not only require complete dissolution after fracturing is completed but also require a certain degree of strength and plasticity [1,2,3,4]. The addition of functional alloying elements to soluble aluminum alloys improves their properties. Among these, Mg, Ga, Sn, and In cause a negative shift in the potential of the aluminum matrix, which implies an increase in the solubility of the soluble aluminum alloys [5,6,7,8,9,10]. Ga is the main element that increases the dissolution performance of soluble aluminum alloys, causing a large degree of negative shift in potential [11,12], and can play a role in refining the effect of grains so that the alloy is uniformly dissolved [13]. Sn forms a second phase with the aluminum matrix embedded in the surface of the oxide film, blocking the continuity of the oxide film and thus forming a channel for the exchange of substances between the matrix and the outside world, increasing the corrosion rate and improving the dissolution rate [14,15,16]. Elements mainly in the matrix and oxide film, between the formation of enriched areas, or the formation of a second phase distributed between the oxide film and the matrix, block the contact between the oxide film and the matrix, thereby increasing the activity of the soluble aluminum alloy and improving the dissolution rate, resulting in a potential negative shift [17,18]. The addition of Mg can influence the matrix plasticity [19] and other mechanical properties, and the appropriate amount of addition that can simultaneously form a solid solution with the aluminum matrix and strengthen the solid solution can also refine the grain and synergistically improve the strength of the soluble aluminum alloy.

The final properties of soluble aluminum alloys are affected differently by the type and content of the elements added to the aluminum matrix [20,21,22]. For example, Kulkarni et al. investigated the effect of different levels of Mg on the surface free energy of Al-5Zn-0.03In alloy and found that at 2% Mg, the alloy had the lowest surface free energy, which promoted grain refinement and homogeneous dissolution of the alloy [23]; however, the paper did not mention the effect on the plasticity of the alloy. Hamed explored the solubility of soluble aluminum alloys from the machining process and analyzed the effect of aging heat treatment on the organizational structure and corrosion behavior of friction surfacing Al-Zn-Mg-Cu alloys. The results showed that after 18 h of post-treatment with artificial aging heat treatment at 125 °C, significant grain growth was observed in the nickel-free samples. The shear strength and hardness of the nickel-containing samples increased by 24% and 13%, respectively, compared with those of the aluminum matrix, but the corrosion decreased by 39% compared to that of the Ni-free sample [4].

Therefore, this study conducts an in-depth analysis of aluminum alloys with different Mg contents from the aspects of mechanical properties, solubility, electrochemical properties, microscopic morphology, and composition. Based on the optimum solubility, this study focuses on exploring the optimum Mg content ratio for alloy properties.

## 2. Materials and Methods

### 2.1. Preparation of Al Alloy

The Al-Mg-Ga-Sn-In alloy was cast using raw materials of pure Al (99.95 wt.%), pure Mg (99.95 wt.%), pure Ga (99.99 wt.%), pure Sn (99.99 wt.%), and pure In (99.99 wt.%), in a melting furnace around 720 °C−760 °C by graphite crucible. After holding for 30 min, the required low-melting-point and strengthened alloys were added and stirred thoroughly. The temperature was maintained for 30 min before adding Al-Ti-B intermediate alloy refining and slag removal agents. After 15 min, degassing was performed and the melted alloy was poured into a preheated cylindrical steel mold at 150 °C.

**Figure 1:**
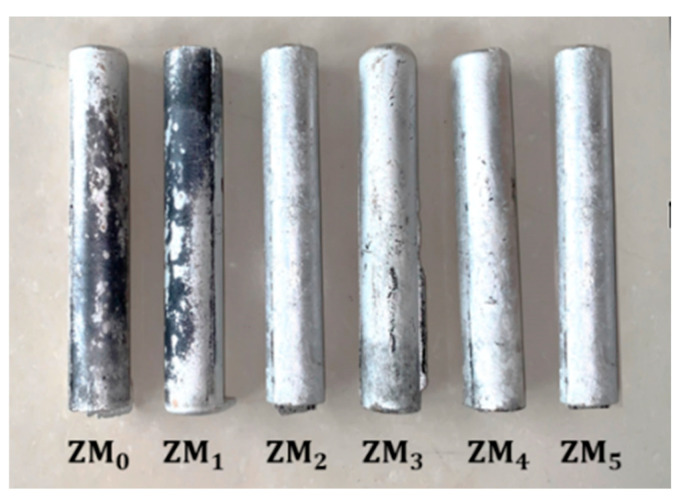
Cylindrical ingots of Al-Mg-Ga-Sn-In alloys with different Mg contents.

The cast Al-Mg-Ga-Sn-In alloy was a cylindrical ingot with dimensions of φ40 mm × 300 mm, as shown in Figure 1. Among the elements, the Ga:Sn ratio was 1, with a content of 1.0 wt.%, Mg content was between 0 wt.% and 5.0 wt.%, and the mass fraction of the low melting point metal In was 0.7 wt.%. The specific composition of the alloy is shown in Table 1.

### 2.2. Microstructure Observation and Composition Analysis

#### 2.2.1. Metallographic Organization and Surface Topography

The specimen was sanded, polished, and corroded for observation using an S-4800 scanning electron microscope (SEM; Nippon Electronics Corporation, Minato City, Japan). For grinding the sample, 800 mesh sandpaper was used to coarsely grind the sample to obtain a relatively flat surface, 2000 mesh sandpaper was used to finely grind the sample, and emery polishing paste was used to polish the sample to remove the surface wear marks. After polishing, the surface was cleaned with absolute ethanol and dried for corrosion treatment. Using the chemical corrosion method (Keller corrosive agent content of each component was 1 mL HF: 1 mL HNO_3_:95 mL H_2_O), a cotton swab was dipped in a corrosive agent to apply flat on the polished surface; after 1–2 s the polished surface changed slightly, was immediately washed with absolute ethanol, dried, and then used to observe the tissue morphology.

The surface morphology, second-phase morphology, tensile fracture morphology, dissolution process, and final product morphology of the soluble aluminum alloy were observed using electron microscopy.

#### 2.2.2. Composition Analysis

The content and distribution of each element in the soluble aluminum alloy were analyzed using an S-4800 scanning electron microscope (SEM) with an energy dispersive X-ray spectrometer (EDS) and electron backscatter diffraction (EBSD).

A D/max2200PC X-ray diffractometer (Japan Science and Technology Co., Ltd., Tokyo, Japan) was used for material phase analysis, and the lattice spacing, diffraction intensity, and diffraction angle were measured. The main parameters used in this study were a voltage of 35 KV, current of 25 mA, sampling time of 0.5 s, and scanning start angle and end angle of 10° and 90°, respectively. The test specimen of Al-Mg-Ga-Sn-In soluble aluminum alloy was a φ15 mm×5 mm cylinder, and the test surface was a polished flat surface.

### 2.3. Tensile and Compressive Performance Test

**Figure 2:**
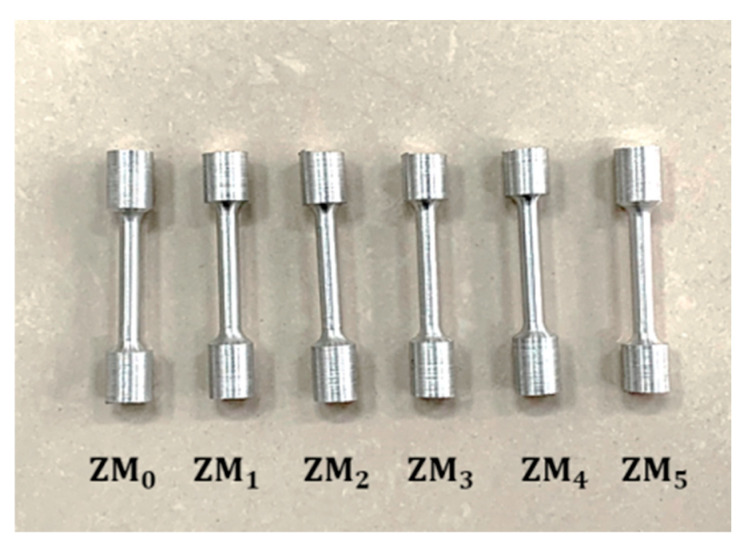
Tensile specimen pieces of Al-Mg-Ga-Sn-In alloys with different Mg contents (mm).

The tensile strength, yield strength, elongation, section shrinkage rate, compressive strength, compression ratio, and section expansion rate of the materials were measured using a WDW-100 universal material testing machine (Taiwan St Instrument Co., Ltd., Taipei City, Taiwan). Before the tensile and compressive tests, it was necessary to remove the stains and grinding machine marks on the surface of the sample, whereas the anti-tensile parts were required to mark the mark distance. The tensile rate was set at 1.0 mm/min. At the same time, in order to reduce experimental error and improve the accuracy of the experimental results, three tensile specimens were selected for each group of samples for testing, Tensile specimen pieces as showing in Figure 2, according to the requirements of GB/T 16865-2023 [Test pieces and methods for tensile test for wrought aluminium, magnesium and their alloy products] [24].

### 2.4. Hardness Test

A Phase II 900–355 digital display electronic Brinell hardness tester was obtained from Shanghai Jiecun Instrument Equipment Co., Ltd. (Shanghai, China). Brinell hardness (HB) is a commonly used method to measure the hardness of materials: through a certain specification of the test load press a hard steel ball or alloy ball to the surface of the sample, which will leave an indentation; by measuring the length, width and other dimensions of the indentation, calculate the corresponding hardness. The smaller the Brinell hardness value, the softer the material and the larger the indentation diameter.

Before testing, the test surface was flat, and the surface of each sample was randomly measured seven times to obtain a more accurate hardness value. The distance between each indentation was no less than 0.2 mm, and the final hardness was averaged.

### 2.5. Dissolution Performance Test

An HH-8 thermostatic water bath manufactured by Jiangsu Dongpeng Instrument Manufacturing Co., Ltd. (Nantong, China), was used to measure solubility. In this study, a φ35mm ball was used as the dissolution test material, and the dissolution test was carried out in a water bath at a constant temperature of 90 °C. The soluble ball was weighed, the size of its appearance was measured every 1.0 h, and records were taken until the soluble ball was completely dissolved. The formula for calculating the dissolution rate used in this study is shown in Formula (1).

*V* = (*D*_1_ − *D*_0_)/*T* (1)

In the formula:

*V* = Dissolution rate of soluble aluminum alloy in clean water (mm/h);

*D*_1_ = Diameter of soluble sphere before dissolution (mm/h);

*D*_0_ = Diameter of soluble sphere after dissolution (mm/h);

*T* = Dissolution time (h)

## 3. Results and Discussion

### 3.1. Microstructural Observation and Compositional Analysis

#### 3.1.1. SEM and EDS

**Figure 3:**
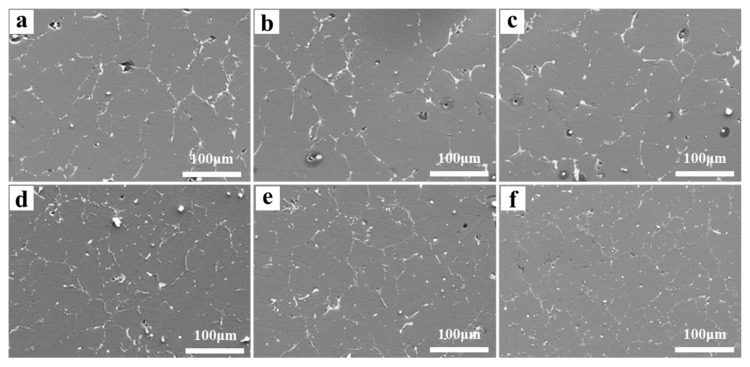
SEM images of the surface of as-cast Al-Mg-Ga-Sn-In soluble aluminum alloys with different Mg contents: (**a**) 0 wt.%, (**b**) 1.0 wt.%, (**c**) 2.0 wt.%, (**d**) 3.0 wt.%, (**e**) 4.0 wt.%, (**f**) 5.0 wt.%.

SEM images of the as-cast Al-Mg-Ga-Sn-In soluble aluminum alloys with different Mg contents are shown in Figure 3. The Al grain structure of the soluble aluminum alloy showed an equiaxed shape, indicating that the Al grain structure of the soluble aluminum alloy grew uniformly during the casting solidification process. As the Mg content increased from 0 wt.% to 3.0 wt.%, the grain size of Al decreased, indicating that Mg played a role in grain refinement. This is because the addition of the Al-Ti-B intermediate alloy refining agent during the casting process is beneficial for generating the TiAl_3_ phase, which can promote the non-uniform nucleation of Al grains and achieve the effect of refining grains [25,26]. When the Mg content increased from 3.0 wt.% to 5.0 wt.%, the grain size of Al gradually increased, indicating that when the Mg content was too high, the degree of undercooling decreased during the casting process, causing compositional undercooling, suppressing the nucleation rate, promoting the growth rate of Al grains, and resulting in coarse grains.

**Figure 4:**
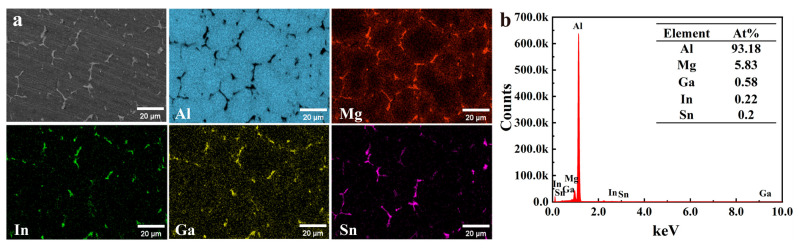
EDS energy spectra of the surface of cast Al-Mg-Ga-Sn-In soluble aluminium alloy with 3.0 wt.% Mg content: (**a**) SEM image and element mapping of the surface, (**b**) Elemental composition chat from surface scanning.

Figure 4 shows the surface SEM image and EDS spectrum of the cast Al-Mg-Ga-Sn-In soluble aluminum alloy with 3.0 wt.% magnesium content. From the surface scanning energy spectrum of the entire region, the main elements include Al, Mg, Ga, In, and Sn; it can be clearly seen from the figure that the second phase is evenly distributed, and its shape is mainly dendritic with small blocks. The surface scanning data show that the main matrix is an Al matrix, while magnesium, gallium, indium, and tin are concentrated in the second phase, distributed around the grain–white grain boundaries. Mg and Ga are evenly distributed in the second phase, whereas In and Sn are partially absent. Further proof is required from the combined phase analysis results. This indicates that some Mg, Ga, and Sn atoms may have precipitated at the grain boundaries as a second phase during the casting process.

#### 3.1.2. EBSD

**Figure 5:**
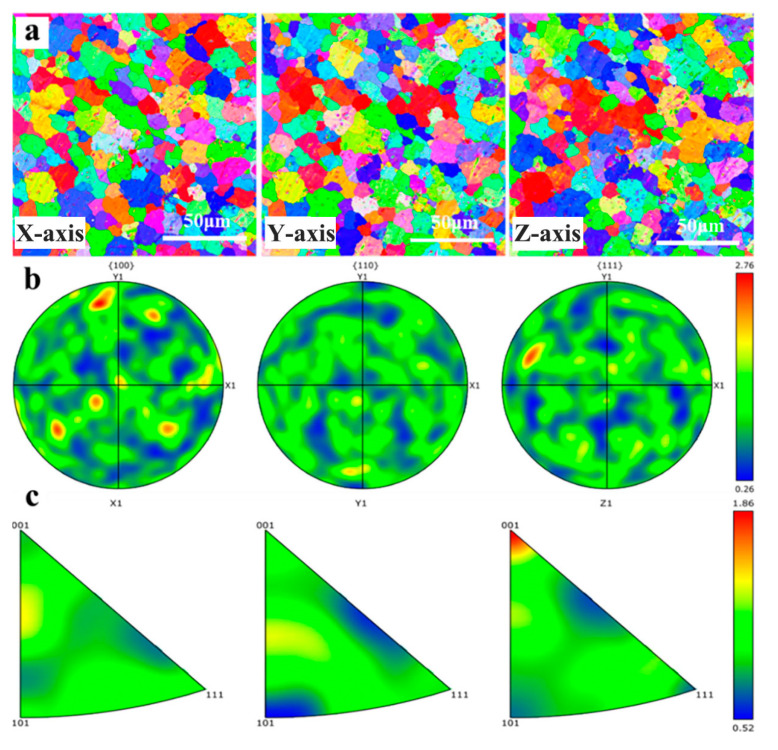
EBSD analysis of the surface of as-cast Al-Mg-Ga-Sn-In soluble aluminum alloy with an elemental Mg content of 3.0 wt.%: (**a**) Histogram of grains with different axes, (**b**) Polar plot, (**c**) Antipolar plot.

**Figure 6:**
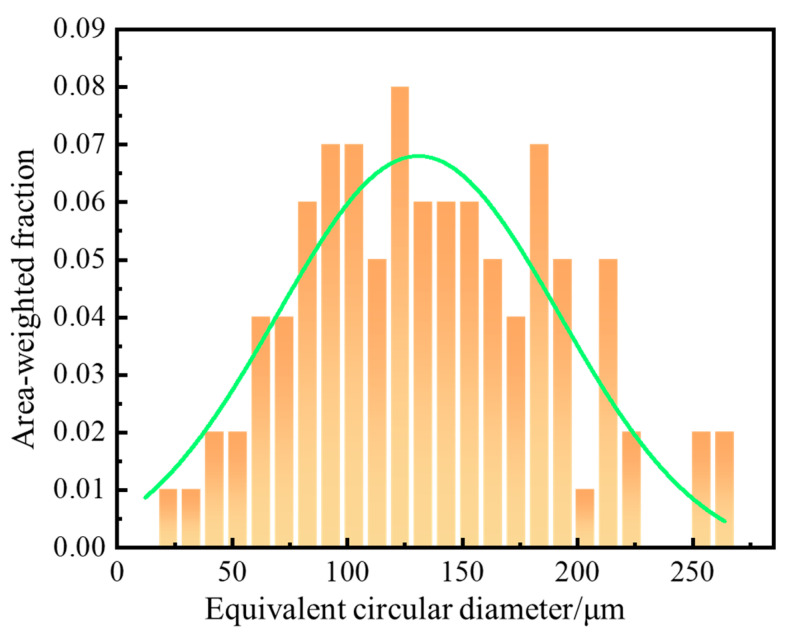
Grain size of as-cast Al-Mg-Ga-Sn-In soluble aluminum alloy with 3.0 wt.% elemental Mg content.

The EBSD analysis of the as-cast Al-Mg-Ga-Sn-In soluble aluminum alloy with 3.0 wt.% elemental Mg is shown in Figure 5. The X-, Y-, and Z-grain structures are shown in Figure 5a. It can be seen from the grain structure diagrams of different axial directions that the grain structure is uniform and the grain size difference in the three axial directions is small. The pole figure shown in Figure 5b indicates significant texture variation along the X- and Z-axes. The inverse pole figure shown in Figure 5c indicates significant texture variation along the Z-axis. The results showed that the Mg content caused a change in the texture orientation, and a smaller change in texture orientation led to a more uniform grain structure size. The grain size diagram of the as-cast Al-Mg-Ga-Sn-In soluble aluminum alloy with a Mg content of 3.0 wt.% is shown in Figure 6. According to the statistics, the average size of the grain structure is 66.7 μm in diameter; the 95% confidence interval is [61.38, 71.96].

#### 3.1.3. Alloy Cross-Section Topography Analysis

**Figure 7:**
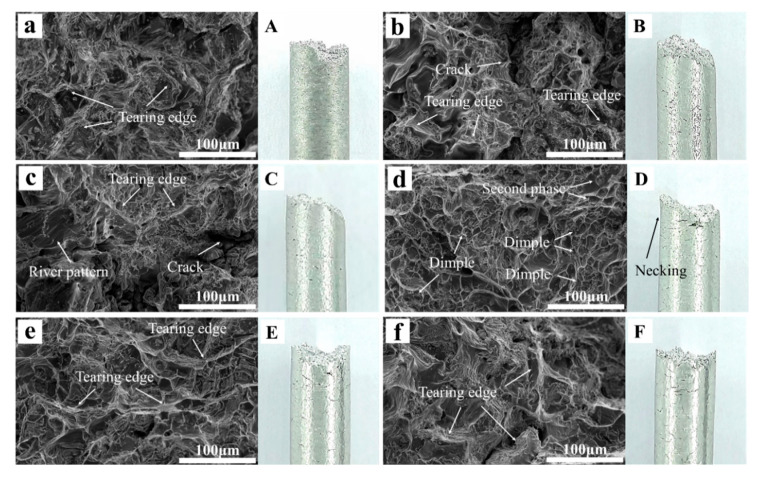
Tensile fracture micrograph (**a**–**f**) and Macro diagram (**A**–**F**) of as-cast Al-Mg-Ga-Sn-In soluble aluminum alloys with different Mg contents: (**a**,**A**) 0 wt.%, (**b**,**B**) 1.0 wt.%, (**c**,**C**) 2.0 wt.%, (**d**,**D**) 3.0 wt.%, (**e**,**E**) 4.0 wt.%, (**f**,**F**) 5.0 wt.%.

Figure 7 illustrates the tensile fracture microstructures of the cast Al-Mg-Ga-Sn-In alloys with different Mg concentrations. Bright white tearing prisms (indicative of brittle fracture characteristics) appeared differently in different specimens. Specifically, alloys ZM_0_, ZM_1_, ZM_2_, ZM_4_, and ZM_5_ exhibited more tear ribs, indicating relatively low plasticity. In contrast, ZM_3_ showed a large number of tough nests, which are distinctive micromorphological features of plastic materials and are caused by plastic deformation, supercooling nucleation, void growth, and coalescence. The morphology is a significant microscopic feature of plastic materials. After the plastic deformation of the material, the supercooling nucleation and growth were aggregated together in the cavity morphology, when the material fractured, at the fracture interface to produce traces left behind. The formation of tough nests at the time of fracture is due to the plastic deformation, which consumes a lot of the work done by the external force; the higher the number of tough nests, the higher its plasticity. The results show that with increasing Mg content, the plasticity of the material increases and then decreases; however, when the Mg content is 3.0 wt.%, examining the microscopic characteristics of the plastic fracture ligamentous fossa, the plasticity is higher because in the casting process of 3.0 wt.% Mg content is conducive to the nucleation, growth, and aggregation of ligamentous fossa. The excessive precipitation will be in the second phase, and most of the second phase of the metal belongs to the hard and brittle phase, which reduces the plasticity.

**Figure 8:**
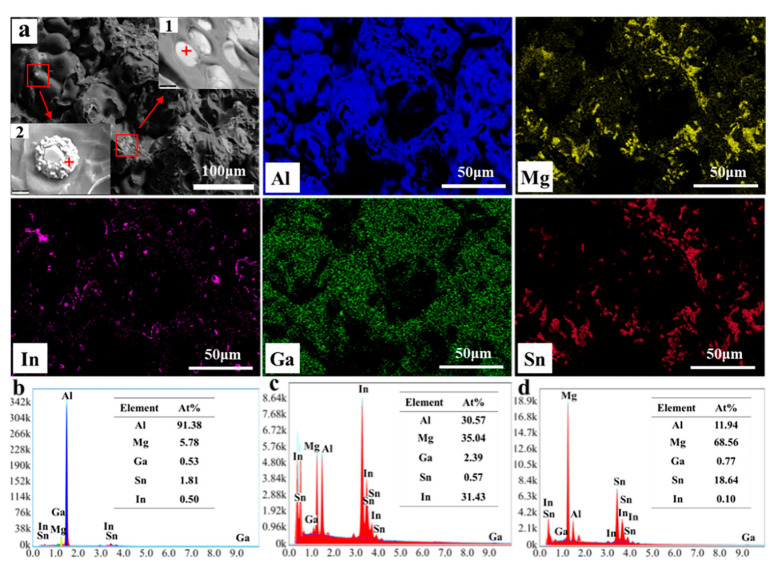
EDS energy spectra of tensile fracture of as-cast Al-Mg-Ga-Sn-In soluble aluminum alloy with an elemental Mg content of 3.0 wt.%: (**a**) SEM image and element mapping of the fracture, (**b**) Elemental composition chart from surface scanning, (**c**) Elemental composition at point 2 (“+” is the collection point), (**d**) Elemental composition at point 1 (“+” is the collection point).

Figure 8 illustrates the histomorphology and energy dispersive spectroscopy (EDS) spectra of the tensile fracture in the cast Al-Mg-Ga-Sn-In soluble aluminum alloy containing 3.0 wt.% elemental magnesium. The surface scanning spectral analysis of the fracture (Figure 8b) indicates that the fracture predominantly comprises Al, Mg, Ga, In, and Sn, with the aluminum matrix phase constituting the majority. In Figure 8a, a small number of gray regions are observed to be interspersed within the dark aluminum matrix. Upon magnifying the field of view, these gray regions exhibited two distinct morphologies: raised particles and notched inlays. Scanning spectral analysis of these gray areas revealed that the compositions of points 1 (notched inlays) and 2 (raised particles) differed slightly. Point 2 (Figure 8c) was primarily composed of Al, Mg, and In, accompanied by minor amounts of Ga and trace Sn. In contrast, Point 1 (Figure 8d) was predominantly composed of Mg, Sn, and Al, with only trace quantities of Ga and In. It was initially inferred that the Mg_2_Sn phase (Figure 8c) and the Ga_2_Mg phase (Figure 8d) might exist in a gray area, primarily located at the grain boundaries [27,28,29]. Both phases exhibited irregular shapes and coexisted with the grain boundaries, making it challenging to differentiate them based solely on their morphology. However, the colors of the two phases were slightly distinct, with the Mg_2_Sn phase appearing dark gray and the Ga_2_Mg phase appearing bright gray. The surface scanning energy spectroscopy data from the fracture indicated that the Ga content was lower than that of Sn. This observation suggests that the bonding energy of the Ga-Mg chemical bond exceeds that of the Mg-Sn bond, facilitating the formation of the Mg_2_Sn phase. Preliminary evidence for the presence of this second phase was provided by the SEM and energy-dispersive EDS analyses. However, accurate identification of the second phase requires corroboration with the results of the XRD analysis.

### 3.2. Phase Analysis

**Figure 9:**
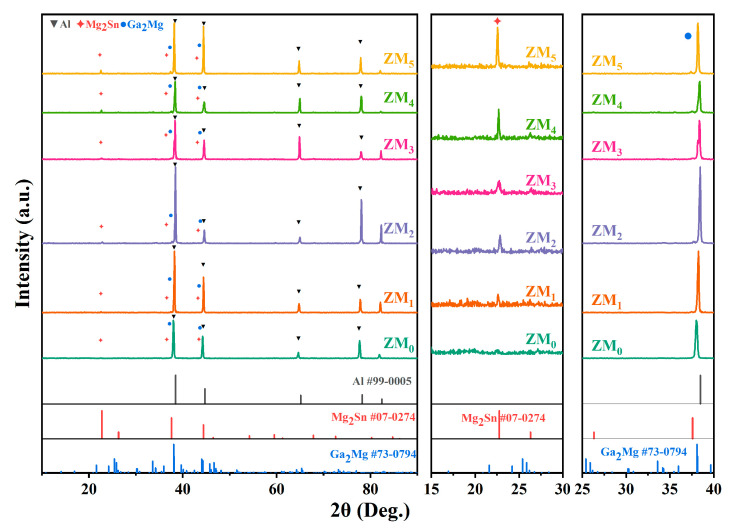
XRD patterns of as-cast Al-Mg-Ga-Sn-In soluble aluminium alloys with different Mg contents before dissolution: (ZM_0_) 0 wt.%, (ZM_1_) 1.0 wt.%, (ZM_2_) 2.0 wt.%, (ZM_3_) 3.0 wt.%, (ZM_4_) 4.0 wt.%, (ZM_5_) 5.0 wt.%.

The XRD patterns of the as-cast Al-Mg-Ga-Sn-In soluble aluminum alloys with different Mg contents before dissolution are shown in Figure 9. It was observed that all alloys contained a matrix α-Al primary phase, Mg_2_Sn intermetallic phase, and Ga_2_Mg intermetallic phase. The XRD patterns show that the diffraction angles (2θ) of the Mg_2_Sn intermetallic phase (111), (220), and (422) peaks vary in the ranges of 22.5°–22.9°, 37.3°–37.7° and 67.6°–68.1°, respectively. The diffraction angles (2θ) of the Ga_2_Mg intermetallic phase (012), (110), (020), and (022) peaks vary in the ranges 34.9°–35.3°, 41.4°–41.8°, 48.2°–48.6° and 55.2°–55.6°, respectively. 

The peak strengths of the second phases, Mg_2_Sn and Ga_2_Mg, simultaneously increased and then decreased with increasing Mg content, indicating that the content of the two intermetallic phases increased with increasing Mg content in the alloy. The peak intensities of the Mg_2_Sn and Ga_2_Mg intermetallic phases were strongest when the Mg content was 3.0 wt.%, indicating that the two intermetallic phases were the most abundant. Meanwhile, with the increase in Mg content, the diffraction peak shifted to the right and then to the left, and when the Mg content was 3.0 wt.%, the diffraction peak was located on the rightmost side, indicating that the Ga, Sn and Mg elements entered the Al matrix lattice instead of the Al atoms to form the replacement solid solution when the soluble aluminum alloys with different contents (0–5.0 wt.%) of Mg were added. However, the atomic diameter of Al is between those of Ga and Sn. Therefore, the Mg atoms make the Al matrix lattice reduce the size of the Al matrix lattice to produce distortion; the lattice spacing is minimized and the grain size is relatively small. The variation in the diffraction angle (2θ) of the Al (111), (200), (220), (311), and (222) peaks and the corresponding peak intensity magnitude indicated that the Al matrix phase changed with the Mg content.

### 3.3. Mechanical Performance Analysis

**Figure 10:**
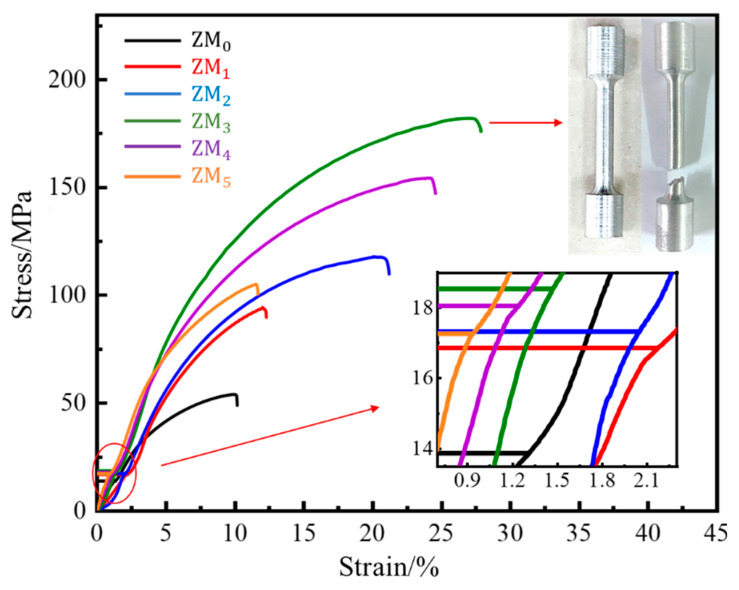
Tensile stress–strain curves of as-cast Al-Mg-Ga-Sn-In soluble aluminium alloys with different Mg contents at room temperature: (ZM_0_) 0 wt.%, (ZM_1_) 1.0 wt.%, (ZM_2_) 2.0 wt.%, (ZM_3_) 3.0 wt.%, (ZM_4_) 4.0 wt.%, (ZM_5_) 5.0 wt.%.

The room-temperature tensile stress–strain curves of cast Al-Mg-Ga-Sn-In soluble aluminum alloys with different Mg contents are shown in Figure 10, and the tensile stress–strain test data are shown in Table 2. From the tensile stress–strain curve, it can be seen that with increasing Mg content, the compressive strength first increased and then decreased, indicating that when the Mg content was too small, the solid solution effect was poor, the grain organization was large, and there were many casting-induced defects, such as voids. As the Mg content continued to increase, the solid solution of Mg increased, and the organization of the defects within the organization was reduced; however, when the Mg content was too high, the organization became oversaturated. The second phase of intermetallic compounds was precipitated. When the Mg content reached 3.0 wt.%, the comparison of specimen ZM_3_ before and after stretching is shown in Figure 10. The tensile strength (σ_b_) was 181.99 MPa, elongation (δ) was 27.49%, and sectional shrinkage (φ) was 11.67%.

**Figure 11:**
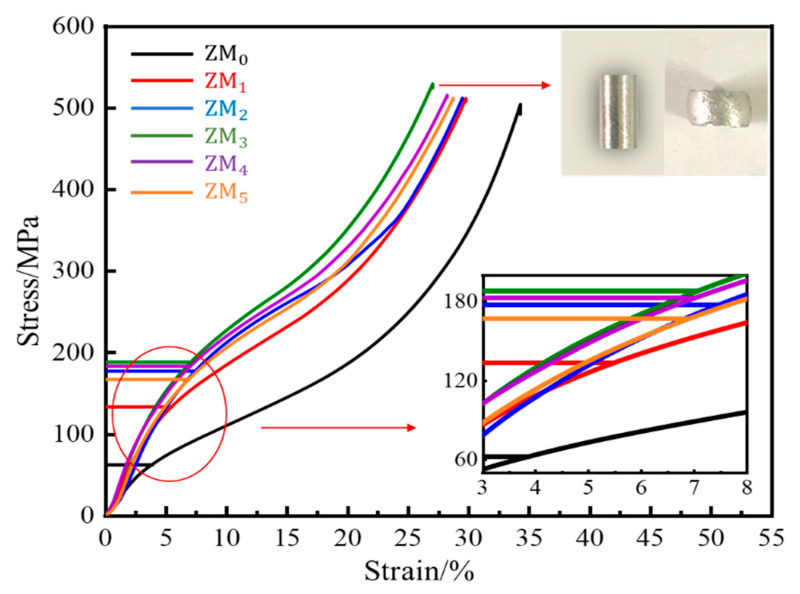
Resisted compressive stress–strain curves of as-cast Al-Mg-Ga-Sn-In dissolvable aluminum alloys with different Mg contents at room temperature: (ZM_0_) 0 wt.%, (ZM_1_) 1.0 wt.%, (ZM_2_) 2.0 wt.%, (ZM_3_) 3.0 wt.%, (ZM_4_) 4.0 wt.%, (ZM_5_) 5.0 wt.%.

From the compressive stress–strain curve, it can be seen that after a certain strain, the specimen continued to deform without fracture as the pressure increased, showing obvious plastic deformation, proving that it is a plastic material. With the increasing Mg content, the compressive yield strength first increased and then decreased, and was relatively high when the Mg content is 3.0 wt.%. A comparison of specimen ZM_3_ before and after compression is shown in Figure 11. The compressive yield strength (σ_sc_) was 188.32 MPa, compression rate (δ) was 27.06%, and section expansion rate (φ) was 138.66%.

**Figure 12:**
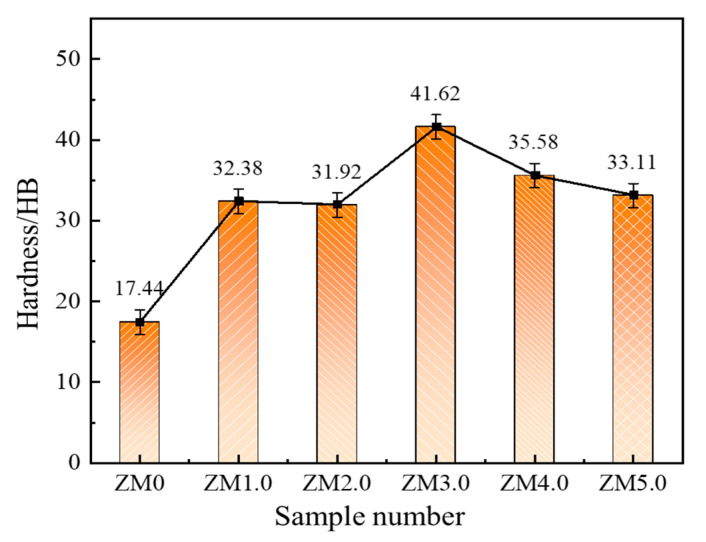
Hardness profiles of as-cast Al-Mg-Ga-Sn-In soluble aluminum alloys with different Mg contents: (ZM0) 0 wt.%, (ZM1) 1.0 wt.%, (ZM2) 2.0 wt.%, (ZM3) 3.0 wt.%, (ZM4) 4.0 wt.%, (ZM5) 5.0 wt.%.

The hardness curves of the as-cast Al-Mg-Ga-Sn-In soluble aluminum alloys with different Mg contents are shown in Figure 12. With the continuous increase in Mg content, the hardness first increased and then decreased, indicating that with the continuous increase in Mg content, the solid solution effect was obvious, and the grain refinement was large; thus, the hardness increased, and when the Mg content was 3.0 wt.%, the hardness reached a maximum of 41.62 HB. However, as the Mg content continued to increase, owing to supersaturation precipitating an excess of the second phase, the second phase of the metal mostly belonged to the hard and brittle phases, and its distribution changed the grain organization, causing a decrease in hardness.

### 3.4. Solubility Performance Analysis

**Figure 13:**
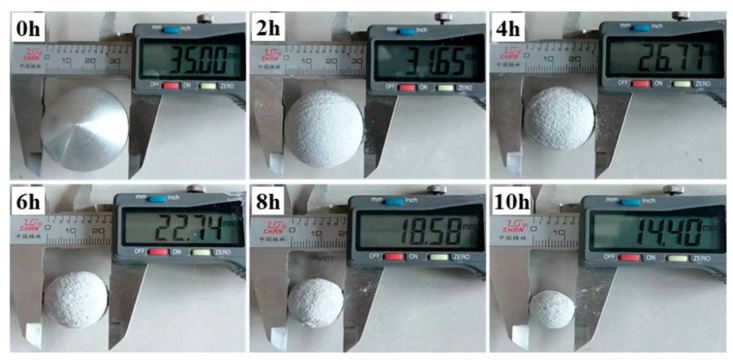
Dissolution diagram of as-cast Al-Mg-Ga-Sn-In soluble aluminum alloy spheres with an elemental Mg content of 3.0 wt.% in water at 90 °C.

**Figure 14:**
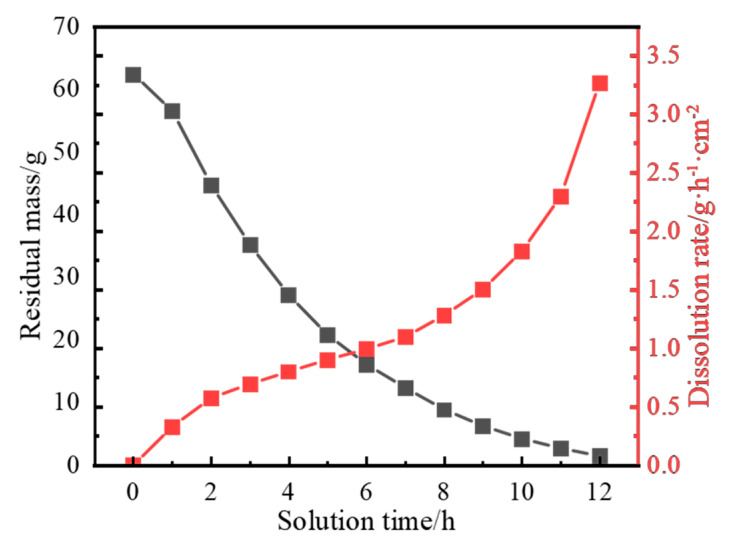
Dissolution residual mass and dissolution rate profiles of as-cast Al-Mg-Ga-Sn-In soluble aluminum alloy spheres with an elemental Mg content of 3.0 wt.% dissolved in water at 90 °C.

A dissolution diagram of cast Al-Mg-Ga-Sn-In soluble aluminum alloy spheres with an elemental Mg content of 3.0 wt.% dissolved in water at 90 °C is shown in Figure 13. The dissolution of cast Al-Mg-Ga-Sn-In soluble aluminum alloy spheres with an elemental Mg content of 3.0 wt.% at 90 °C in water and the remaining mass and rate of dissolution plots are shown in Figure 14.

During the dissolution process, the measurements were recorded at 1.0 h intervals, and it was observed that the volume shape of the sample sphere during the dissolution process was close to that of a regular sphere and changed from large to small, indicating that the dissolution process was uniform. The dissolution residual mass curve is a nonlinear curve, indicating that the dissolution rate was not fixed but changed with the change in dissolution. From the dissolution rate diagram, the dissolution rate increased significantly within 0–3 h, the variation range was in the range of 0–0.7 g·h^−1^cm^−2^, the dissolution rate curve was flat in the range of 3.0–8.0 h, the dissolution rate increased slowly, the change rate was in the range of 0.7–1.2 g·h^−1^cm^−2^; the dissolution rate increased rapidly at 8.0–12.0 h, and the change rate was in the range of 1.2–3.3 g·h^−1^cm^−2^.

## 4. Conclusions

The Al-Mg-Ga-Sn-In soluble aluminum alloy material with sample number ZM_3_ prepared by the high-temperature melting method was formulated with a Mg content of 3.0 wt.%, Ga, Sn content of 1.0 wt.%, and In content of 0.7 wt.%.

Al-Mg-Ga-Sn-In soluble aluminum alloy is a plastic material. When the Mg element was 3.0 wt.%, the tensile strength σ_b_ was 181.99 MPa, the elongation δ was 27.49%, the section shrinkage φ was 11.67%, the compressive yield σ_sc_ was 188.32 MPa, the compressive δ was 27.06%, and the section elongation φ was 138.66%. Mg_2_Sn and Ga_2_Mg intermetallic compounds were formed between the functional alloying elements Ga, Sn, and Mg, most of which were distributed at grain boundaries and a few of which were distributed in grains. At the same time, it could be spontaneously dissolved in 90 °C water, with an average dissolution rate of 1.0 g·h^−1^cm^−2^ and a maximum dissolution rate of 3.25 g·h^−1^cm^−2^ during dissolution for 12.0 h.

## Figures and Tables

**Table 1:** Formulation for casting Al-Mg-Ga-Sn-In soluble aluminium alloy ingots with different Mg contents.

Sample Number	Elements and Content of Each Element (wt.%)			
	Mg	Ga	Sn	In
ZM_0_	0	1.0	1.0	0.7
ZM_1_	1.0	1.0	1.0	0.7
ZM_2_	2.0	1.0	1.0	0.7
ZM_3_	3.0	1.0	1.0	0.7
ZM_4_	4.0	1.0	1.0	0.7
ZM_5_	5.0	1.0	1.0	0.7

**Table 2:** Table of data for tensile stress–strain tests of as-cast Al-Mg-Ga-Sn-In soluble aluminum alloys with different Mg contents at room temperature.

Sample Number	σ_b_/MPa	σ_s_/MPa	δ/%	φ/%
ZM_0_	53.916 ± 0.85	13.866 ± 0.50	10.06 ± 0.90	4.72 ± 1.10
ZM_1_	94.357 ± 1.30	16.863 ± 0.90	12.17 ± 0.50	5.89 ± 0.50
ZM_2_	117.883 ± 1.30	17.319 ± 1.00	20.77 ± 0.30	7.84 ± 0.50
ZM_3_	181.989 ± 1.60	18.541 ± 0.80	27.49 ± 0.30	11.67 ± 0.50
ZM_4_	154.383 ± 1.30	18.051 ± 1.00	24.31 ± 0.50	8.92 ± 0.50
ZM_5_	105.078 ± 1.20	12.267 ± 0.80	11.63 ± 0.70	6.42 ± 0.90

The room-temperature compressive stress–strain curves of cast Al-Mg-Ga-Sn-In soluble aluminum alloys with different Mg contents are shown in Figure 11, and the compressive stress–strain test data are shown in Table 3.

**Table 3:** Table of data for compressive stress–strain tests of as-cast Al-Mg-Ga-Sn-In soluble aluminum alloys with different Mg contents at room temperature.

Sample Number	σ_b_/MPa	σ_sc_/MPa	δ/%	φ/%
ZM_0_	504.12 ± 1.00	62.65 ± 0.60	34.28 ± 0.90	201.32 ± 1.10
ZM_1_	511.46 ± 1.00	133.84 ± 1.00	29.73 ± 0.50	183.69 ± 1.10
ZM_2_	511.74 ± 1.10	177.53 ± 1.10	29.47 ± 0.50	174.91 ± 0.80
ZM_3_	529.20 ± 0.80	188.32 ± 1.00	27.06 ± 0.70	138.66 ± 1.00
ZM_4_	515.29 ± 1.00	183.06 ± 1.00	28.24 ± 0.60	164.20 ± 1.00
ZM_5_	511.51 ± 1.00	167.09 ± 1.00	28.78 ± 0.60	165.52 ± 1.00

## Data Availability

The original contributions presented in the study are included in the article; further inquiries can be directed to the corresponding author/s.
